# Structural and Regulatory Changes in PBP4 Trigger Decreased β-Lactam Susceptibility in Enterococcus faecalis

**DOI:** 10.1128/mBio.00361-18

**Published:** 2018-04-03

**Authors:** Louis B. Rice, Charlene Desbonnet, Amelia Tait-Kamradt, Monica Garcia-Solache, John Lonks, Thomas M. Moon, Éverton D. D’Andréa, Rebecca Page, Wolfgang Peti

**Affiliations:** a Department of Medicine, Warren Alpert School of Medicine of Brown University, Providence, Rhode Island, USA; b Department of Microbiology and Immunology, Warren Alpert School of Medicine of Brown University, Providence, Rhode Island, USA; c Department of Chemistry and Biochemistry, College of Medicine, University of Arizona, Tucson, Arizona, USA; University of Rochester

**Keywords:** antibiotic resistance, *Enterococcus*, penicillin-binding proteins

## Abstract

Enterococcus faecalis strains resistant to penicillin and ampicillin are rare and have been associated with increases in quantities of low-affinity penicillin-binding protein 4 (PBP4) or with amino acid substitutions in PBP4. We report an E. faecalis strain (LS4828) isolated from a prosthetic knee joint that was subjected to long-term exposure to aminopenicillins. Subsequent cultures yielded E. faecalis with MICs of penicillins and carbapenems higher than those for wild-type strain E. faecalis JH2-2. Sequence analysis of the *pbp4* gene of LS4828 compared to that of JH2-2 revealed two point mutations with amino acid substitutions (V223I, A617T) and deletion of an adenine from the region upstream of the predicted *pbp4* −35 promoter sequence (UP region). Purified PBP4 from LS4828 exhibited less affinity for Bocillin FL than did PBP4 from JH2-2, which was recapitulated by purified PBP4 containing only the A617T mutation. Differential scanning fluorimetry studies showed that the LS4828 and A617T variants are destabilized compared to wild-type PBP4. Further, reverse transcription-PCR indicated increased transcription of *pbp4* in LS4828 and Western blot analysis with polyclonal PBP4 antibody revealed greater quantities of PBP4 in LS4828 than in JH2-2 lysates and membrane preparations. Placing the promoter regions from LS4828 or JH2-2 upstream of a green fluorescent protein reporter gene confirmed that the adenine deletion was associated with increased transcription. Together, these data suggest that the reduced susceptibility to β-lactam antibiotics observed in E. faecalis LS4828 results from a combination of both increased expression and remodeling of the active site, resulting in reduced affinity for penicillins and carbapenems.

## IMPORTANCE

Enterococcus faecalis is an important cause of community-acquired and nosocomial infections and creates therapeutic dilemmas because of its frequent resistance to several classes of antibiotics. We report an E. faecalis strain with decreased ampicillin and imipenem susceptibility isolated after prolonged courses of aminopenicillin therapy for a prosthetic joint infection. Its reduced susceptibility is attributable to a combination of increased quantities of low-affinity PBP4 and an amino acid substitution in proximity to the active site that destabilizes the protein. Our findings provide a cautionary tale for clinicians who elect to “suppress” infections in prosthetic joints and offer novel insights into the interaction of β-lactam antibiotics with low-affinity PBP4. These insights will help inform future efforts to develop therapeutics capable of inhibiting clinical enterococcal strains.

## INTRODUCTION

Enterococcus faecalis is a highly significant pathogen causing bloodstream, soft tissue, and urinary tract infections in hospitalized patients and native and prosthetic valve endocarditis in patients from the community (1). While resistance to penicillin and ampicillin (Amp) is not as significant a problem in E. faecalis as it is in Enterococcus faecium, susceptibilities to penicillins in E. faecalis are less than they are in the streptococci with which they are usually compared (2). *In vitro* data indicate that carbapenems are active against E. faecalis at a level similar to that of penicillins, although clinical experience with these agents is limited (3). In addition to its decreased susceptibility to penicillin and Amp, E. faecalis is fully resistant *in vitro* to most cephalosporins (ceftaroline and ceftobiprole are the exceptions) and tolerant to the bactericidal activity of the few β-lactam antibiotics that are active (2). This tolerance led to the addition of an aminoglycoside to Amp or vancomycin to yield acceptable endocarditis cure rates (4). More recent clinical data indicate that combinations of Amp and ceftriaxone are equivalent to penicillin-aminoglycoside combinations in treating E. faecalis native valve endocarditis (5, 6).

Decreased susceptibility to penicillin and Amp in E. faecalis has been attributed to the activity of a single, low-affinity penicillin-binding protein (PBP) most frequently designated PBP4 but occasionally designated PBP5 (7–9). Strains with reduced levels of penicillin and/or Amp susceptibility have been rarely reported and in some instances have been observed in association with amino acid changes in PBP4. In none of the published studies that we are aware of has there been any work performed to specifically confirm that amino acid substitutions associated with increased penicillin MICs result in a lower affinity for these agents. In the present report, we describe an E. faecalis strain isolated from a prosthetic joint infection that had undergone prolonged β-lactam treatment and was found to have elevated MICs of penicillin, Amp, and imipenem. We present evidence that these elevated MICs are attributable to both decreased penicillin affinity associated with an amino acid substitution in PBP4 and greater quantities of PBP4 due to increased transcription of *pbp4* associated with a point mutation upstream of the putative *pbp4* promoter region.

### Case report.

A middle-aged woman underwent a total knee replacement because of osteoarthritis. The initial prosthesis became infected with methicillin-sensitive Staphylococcus aureus and was replaced in a two-stage procedure in which she received 6 weeks of intravenous cefazolin and 3 months of orally administered ciprofloxacin and rifampin. Shortly thereafter, the knee again appeared inflamed and cultures obtained by incision and drainage grew E. faecalis, followed by a second incision and drainage with a polyethylene tray exchange. During that hospitalization, a total of five cultures grew E. faecalis; the MIC of penicillin was 2 µg/ml for four of the isolates and 4 µg/ml for one of the isolates. She was treated postoperatively with 8 weeks of intravenous Amp with the addition of gentamicin for the first 3 weeks. Sixteen months later, a repeat incision and drainage of the left knee were performed; cultures grew E. faecalis with an MIC of penicillin of 2 µg/ml and Enterococcus durans with an MIC of penicillin of 4 µg/ml. She was suppressed with 500 mg of amoxicillin-clavulanic acid 3 times a day for 8 months, followed by a dose reduction to 500 mg twice a day for another 6 months and finally 500 mg of amoxicillin twice a day for an additional 40 months. Because of continued pain, she underwent removal of the prosthetic knee. Intraoperative cultures grew E. faecalis; the MIC of penicillin was 16 µg/ml, and that of Amp was 8 µg/ml.

## RESULTS

### Susceptibility of E. faecalis strain.

As the original susceptible isolate was not available, we compared the susceptibilities of the clinical isolate (designated E. faecalis LS4828) with those of fully penicillin-susceptible E. faecalis JH2-2 by broth microdilution. MICs for JH2-2 and LS4828 are shown in Table 1. LS4828 exhibited greater susceptibility to penicillin (8-fold MIC increase), Amp (8-fold increase), and imipenem (16-fold increase) than JH2-2. Both were highly resistant to ceftriaxone. E. faecalis LS4828 was tested for β-lactamase activity with nitrocefin disks. Even after prolonged incubation, no activity was detected (data not shown).

**TABLE 1:** MICs of different antibiotics for penicillin-resistant E. faecalis LS4828 and control strains

**Strain**	MIC (μg/ml)^a^					
	**Pen**	**Amp**	**Cro**	**Imp**	**Mer**	**Ery**
LS4828	12.5	12.5	>100	12.5	50	1.56
JH2-2	1.56	1.56	>100	0.78	6.25	0.78
CH19	3.13	0.78	100	0.78	3.13	>100
D366	1.56	0.78	6.25	1.56	3.13	>100

a Abbreviations: Pen, penicillin; Cro, ceftriaxone; Imp, imipenem; Mer, meropenem; Ery, erythromycin.

### *pbp4* from strain LS4828 has two mutations, V223I and A617T, relative to JH2-2.

**FIG 1:**
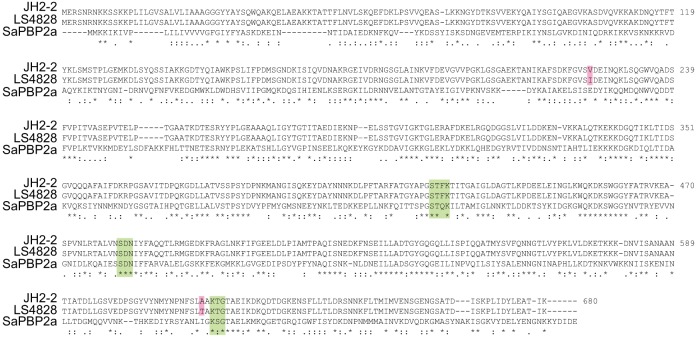
Comparison of PBP4 amino acid sequences of from JH2-2 and LS4828 and alignment with S. aureus PBP2a. Catalytic motifs (green) and sequence differences in PBP4 (red) are highlighted.

To identify the origin of β-lactam resistance in LS4828, the sequence of PBP4 was determined and compared to that of JH2-2. The sequence alignment in Fig. 1 shows a two-amino-acid difference, a valine-to-isoleucine change at residue 223 (V223I) and an alanine-to-threonine change at residue 617 (A617T). The latter mutation is predicted to be close to the known active site of similar PBPs (Fig. 1) (10).

### PBP4 from LS4828 (V223I/A617T) and the A617T variant have lower apparent acylation rates and less affinity for Bocillin FL than PBP4 from JH2-2.

**FIG 2:**
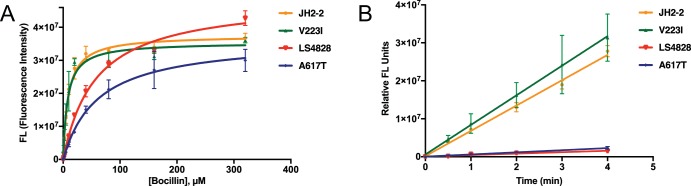
Comparative affinities of PBP4 from JH2-2 and LS4828 and variants thereof. LS4828 PBP4 and the A617T variant show reduced Bocillin FL affinities and apparent acylation rates. (A) Michaelis-Menten (*K*_*m*_) curves were derived from the Bocillin FL binding of each PBP4 enzyme by using SDS-PAGE data and graphing relative fluorescence intensity versus the substrate concentration. Error bars indicate the standard deviation of the mean of two independent experiments. (B) Apparent rates of 10 µM Bocillin FL acylation over time are graphically presented for all PBP4 variants with error bars indicating the standard deviation of the mean of two independent experiments. The slopes of these progress curves were determined in GraphPad Prism version 5 and represent the rate of formation of the stable PBP-Bocillin FL (acyl) complex. The A617T and LS4828 PBP4-Bocillin FL complexes are formed at similar lower rates than those formed by JH2-2 and V223I.

We used Michaelis-Menten kinetics to analyze the activities of PBP4 from JH2-2 (wild type [WT]), LS4828 (V223I/A617T), and the single amino acid variants A617T and V223I as a function of the substrate (Bocillin FL) concentration. For these studies, we used soluble constructs of PBP4 that lack the PBP4 transmembrane domain (PBP4_36-680_; here referred to as PBP4). PBP4s were evaluated throughout the purification steps by SDS-PAGE and determined to be intact and stable after one freeze-thaw cycle prior to use in the Bocillin FL binding experiments (data not shown). The *K*_*m*_ values for the PBP4 variants were determined by using a 0 to 320 µM Bocillin FL concentration range over a 20-min reaction time for two independent experiments (Fig. 2A). The fluorescence intensity values, representing the formation of the stable PBP-Bocillin (acyl) complex, were derived from the SDS-PAGE gels, and the graph shows the mean and standard deviation of each substrate concentration. The data show that the *K*_*m*_ values of the LS4828 and A617T PBP4 variants were 6.4- and 6.8-fold higher, respectively, than those of JH2-2 and V223I (Table 2). To measure the apparent acylation rate (*k*_2_) at a fixed Bocillin FL concentration, early time points of the PBP4 variants and Bocillin FL substrate at subsaturating concentrations were used and the acylation rates were determined by plotting the gel-based fluorescence values against time to compare the slopes of the linear reactions (Fig. 2B). The graph represents the apparent acylation rates in two independent experiments with mean values and standard deviations determined in Excel. The calculated slopes show that the LS4828 and A617T PBP4 variants bind 10 µM Bocillin FL approximately 12- to 18-fold more slowly than the JH2-2 and V223I PBP4 variants (374,000 and 559,600 versus 6,664,000 and 7,790,000, respectively). Together, these kinetic data show that the LS4828 variant has less affinity for penicillin and a lower apparent acylation rate than the WT and further that these changes are due to a single mutation, A617T, in PBP4.

**TABLE 2:** Comparison of Bocillin FL binding affinities for WT JH2-2 and mutant PBP4

**PBP4 enzyme**	***K*_*m*_ (μM)**	***V*****_max_**^a^
JH2-2	9.0 ± 1.0	3.76E+07
LS4828	57.2 ± 5.7	4.86E+07
V223I variant	7.7 ± 1.3	3.54E+07
A617T variant	60.9 ± 4.1	3.65E+07

a Relative fluorescence units.

### The A617T mutation destabilizes the PBP4 fold.

To determine how the A617T mutation affects the stability of PBP4 *in vitro*, we used differential scanning fluorimetry (DSF). The data show that the *T*_*m*_ of PBP4 from LS4828 is more than 6°C lower than that of WT PBP4 (48.2°C versus 54.6°C, respectively; Table 3). Further, the data show that this reduction in thermal stability is due solely to the A617T mutation, as the *T*_*m*_ of PBP4 A617T decreases by nearly the same amount as that of the LS4828 variant, namely, 5.9°C (the PBP4 A617T *T*_*m*_ is 48.7°C). Together, the data show that the A617T mutation destabilizes the PBP4 fold, which most likely leads to a reduced affinity of this variant for antibiotics.

**TABLE 3:** Melting temperatures of WT and variant forms of PBP4

Protein	Mean DSF *T*_*m*_ (°C) ± SD	Δ*T*_*m*_ (°C)
WT from JH2-2	54.6 ± 0.2	
LS4878 (V223I/A617T) variant	48.2 ± 0.2	−6.4
A617T variant	48.7 ± 0.1	−5.9

### LS4828 *pbp4* transcription levels are higher than those of JH2-2 *pbp4*.

**FIG 3:**
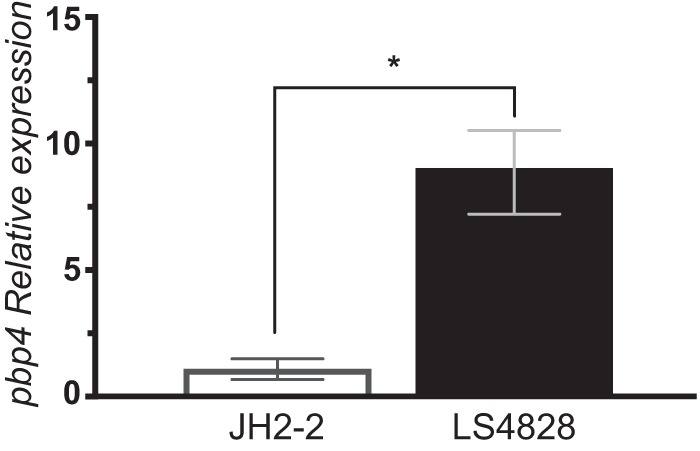
Expression of the *pbp4* gene is higher in penicillin-resistant isolate LS4828 than in the penicillin-sensitive control strain. Cultures were grown in BHI broth to mid-log phase, and cells were harvested and frozen for RNA preparation. 16S rRNA gene expression levels were used as a reference. Error bars indicate the standard deviation of the mean of three independent experiments. *, *P* < 0.0001.

To determine whether increased transcription of *pbp4* contributed to the higher levels of penicillin resistance, quantitative reverse transcription-PCR (RT-qPCR) was carried out with RNAs from LS4828 and JH2-2. Three independent experiments were done, with LS4828 *pbp4* showing an 8.86-fold difference from JH2-2 *pbp4* (Fig. 3).

### A promoter mutation accounts for some or all of the increased transcription of *pbp4* in LS4828.

**FIG 4:**
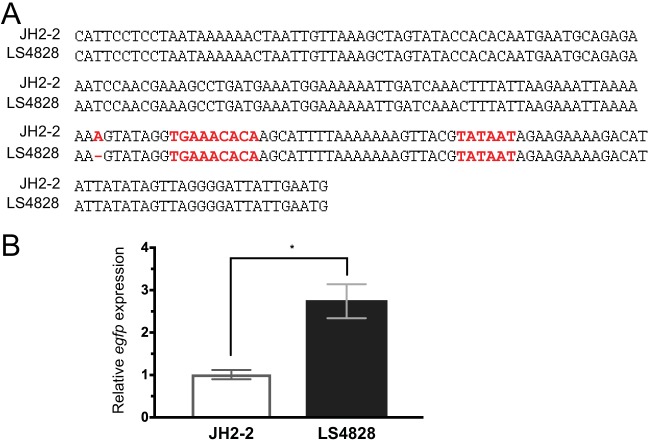
Transcriptional upregulation of *pbp4* in LS4828 is due to a single base deletion in the promoter region of the gene. (A) Sequence comparison of the 200-bp region between *pbp4* and the gene immediately upstream of it. There is a single base change, loss of a single A in a string of seven, upstream of a putative −35 sequence. (B) Relative expression of *egfp* driven by the *pbp4* upstream sequence is higher from the LS4828 promoter than from the JH2-2 promoter. *, *P* < 0.0001.

Sequence analysis of the region upstream of *pbp4* revealed a single base pair deletion 8 bases upstream of a putative −35 region for the *pbp4* gene that consists of the loss of an A residue in a string of seven (Fig. 4A). This region from both JH2-2 and LS4828 was cloned into shuttle vector pBSU101 upstream of the *egfp* (enhanced green fluorescent protein variant gene) coding sequence and expressed in E. faecalis OG1X (11). The upstream region of LS4828 shows significantly greater transcription of the *egfp* reporter than the upstream region of JH2-2 when tested in this context (Fig. 4B).

### LS4828 expresses higher levels of PBP4 than does JH2-2.

**FIG 5:**
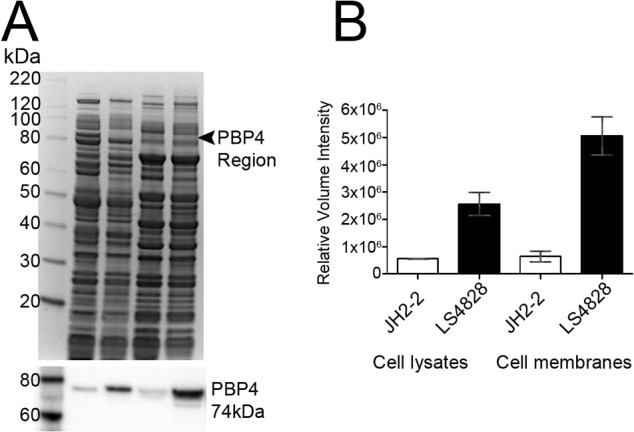
Quantitation of PBP4 in the lysate and membrane fractions of JH2-2 and LS4828 shows increased expression in strain LS4828. (A, top) Coomassie-stained 4 to 12% NuPAGE gel and Western blot analysis of E. faecalis samples. Lanes: 1, MagicMark XP Standards; 2, JH2-2 cell lysate; 3, LS4828 cell lysate; 4, JH2-2 membranes; 5, LS4828 membranes. (A, bottom) Western blot analysis of Coomassie-stained gel (above) with polyclonal PBP4 antibody. (B) Analysis of a Western blot assay by densitometry shows 4- and 7-fold increases in PBP4 expression from LS4828 cell lysates and membranes, respectively, and is representative of two independent experiments.

To determine the PBP4 expression levels of the sensitive JH2-2 and resistant LS4828 E. faecalis strains, we analyzed cell lysates and bacterial membrane preparations by Western blotting. The soluble constructs of *pbp4* (PBP4_36-680_) from JH2-2, LS4828, V223I, and A617T were used as controls in a Western blot analysis of E. faecalis cell lysate and membrane proteins. The purified PBP4s derived from the WT and mutant strains were confirmed by Western blot analyses to bind with equal affinity to the rabbit anti-PBP4 polyclonal antibody (data not shown). Protein loading of all samples was confirmed by Coomassie-stained replicate SDS-PAGE. A comparison of the densitometric data from the autoexposed Western blot showed 4- and 7-fold increases in the expression of PBP4 in strain LS4828 lysate and membrane samples, respectively (Fig. 5A and B). This increased expression of PBP4 in the penicillin-resistant strain was further confirmed in two independent experiments.

## DISCUSSION

Resistance to most β-lactam antibiotics in E. faecalis was attributed to the expression of a low-affinity PBP called PBP4 (although early publications refer to it as PBP5). Early studies indicated that enterococci grew robustly if this low-affinity PBP was <90% saturated, despite the complete saturation of all other identified PBPs (12), and that 90% saturation of PBP5 correlated with the MIC for the strain tested.

Early *in vitro* work on penicillin resistance in E. faecalis revealed a mutant whose resistance was attributed to increased quantities of PBP4, as determined by fluorography with ^125^I-labeled penicillin (7). This mutant, obtained by sequential passage of an E. faecalis strain on progressively increasing concentrations of penicillin, had the same sequences of the *pbp4* promoter region and the structural gene as the parent. The mechanism of increased expression of PBP4 was not identified in that study.

Subsequent work examining clinical strains led to the identification of point mutations in *pbp4* associated with increased MICs of β-lactams. Ono and colleagues (9) reported two clinical strains with increased Amp MICs. The less resistant strains (MIC of 8 µg/ml) had a single P520S amino acid change in PBP4. A more resistant strain (Amp MIC of 16 µg/ml) had H605Y and P520S amino acid changes in PBP4. As these amino acid changes are between the active-site-defining SDN and KTG motifs (Fig. 1), the authors suggested that these variations were likely responsible for the increased MICs. In a subsequent study, Infante and colleagues (8) identified another amino acid variant (D573E) that was associated with a small increase in the penicillin MIC. Neither study fully confirmed the mode of action of these variants.

In contrast to the lack of studies analyzing the effects of structural changes in PBP4 on antimicrobial susceptibility, low-affinity PBP5 from E. faecium has been extensively studied. Rice and colleagues (13) looked at several different point mutations that, collectively, were associated with very high levels of Amp resistance (MICs of >128 μg/ml) and showed that there was a cumulative impact of the different amino acid substitutions. Amino acid changes in E. faecium PBP5 molecules have also been used to help define the lineages of different E. faecium strains (14).

The data presented here confirm, for the first time, the impact of a specific amino acid change in E. faecalis PBP4 on the affinity of the PBP4 molecule for the β-lactam antibiotic Bocillin FL (15). We identified two differences between PBP4 from strain LS4828 and PBP4 from strain JH2-2. One amino acid difference (V223I) occurs in the N-terminal domain of the molecule. This region is not known to have any enzymatic function. The second amino acid difference (A617T) occurs in the transpeptidase domain of PBP4, two amino acids N terminal to the KTG motif, which facilitates β-lactam binding. Purified PBP4 with only the A617T mutation exhibited a reduction in affinity for Bocillin FL that was similar to that observed for PBP4 from LS4828. These findings suggest that the V223I change contributes negligibly to the reduced affinity of PBP4 for β-lactam antibiotics, although it could play a different role, for example, by affecting protein-protein interactions.

**FIG 6:**
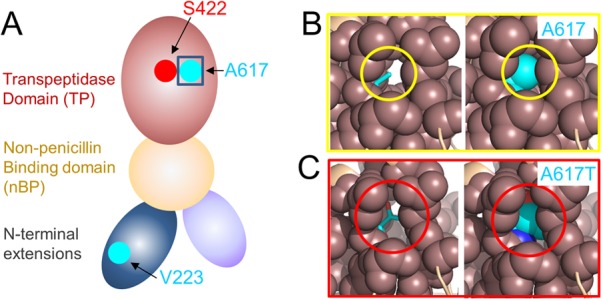
A617 defines the center of an extensive hydrophobic pocket that is adjacent to the PBP4 catalytic pocket. (A) Model of PBP4 based on the domain organization of PBP2a. The locations of the catalytic serine (S424; red) and the two residues mutated in PBP4 from LS4828, V223 and A617 (both cyan), are shown. (B, left) A617 (cyan, sticks) in a model of PBP4 based on the structure of S. aureus PBP2a (PDB code 1MWR; generated with FFAS), with the residues within 5 Å of A617 shown as spheres (coral). (B, right) Same as panel A, except that A617 is shown as spheres. (C, left) PBP4 with the A617T mutation, illustrating the increased space occupied by A617T in this hydrophobic pocket. (C, right) Same as panel B, except that A617T is shown as spheres.

Ala617 is located on the same β-strand as active-site motif III, which includes Thr620, a conserved residue in PBPs that facilitates substrate binding and defines the active-site oxyanion hole. A model of PBP4 based on the structure of PBP2a from S. aureus (PDB code 1MWR) shows that Ala617 defines the center of an extended hydrophobic pocket that is adjacent to the PBP4 active site (Fig. 6). Combined with the thermal stability data, which show that the A617T mutation reduces the PBP4 *T*_*m*_ by 6°C, our model suggests that the increased size and polarity of the Thr side chain in the A617T variant are likely the mechanism by which this pocket and, in turn, the PBP4 transpeptidase domain are destabilized. As a consequence, the *K*_*m*_s of PBP4 of LS4828 and PBP4 A617T for benzylpenicillin derivatives are greater than those of WT PBP4, consistent with the observation that the destabilization leads to a less competent β-lactam binding pocket.

Our data indicate that the clinical strain (LS4828) also expresses larger amounts of PBP4 because of a deletion upstream of the putative −35 region of the *pbp4* promoter. The single adenine deletion falls in a location referred to as the UP region in Escherichia coli promoters (16). The sequences of these regions, which are commonly A-T rich, can have an impact on the expression of downstream genes. Clinical strain LS4828 produces 8.86-fold more *pbp4* transcript than susceptible strain JH2-2. We showed that when the LS4828 promoter was placed upstream of an *egfp* gene and expressed from a plasmid in E. faecalis OG1X, it was associated with a 2.74-fold increase in *egfp* transcript compared to the JH2-2 promoter. These data support the involvement of this adenine deletion in the increased expression of PBP4 in LS4828. The fact that the relative difference in the expression of *egfp* from the two promoters was narrower than the difference in *pbp4* transcription between LS4828 and JH2-2 likely reflects the fact that *egfp* transcription was tested in a different background and the *egfp* gene was expressed from a plasmid that existed in more than a single copy. In any case, the importance of this increase in transcription is supported by the fact that increased quantities of PBP4 were confirmed by Western blotting of whole-cell and membrane lysates of JH2-2 and LS4828 with a polyclonal antibody raised against E. faecalis PBP4.

Querying the genome database for E. faecalis sequences revealed that some strains contain the adenine deletion in the *pbp4* UP region. Most of these strains were from a single project entitled “Penicillin-Resistant Enterococcus faecalis in Poland,” which is to date unpublished. None of the Polish strains contained either the V223I or the A617T substitution; one did have an A618G substitution. The V223I mutation is present in other E. faecalis strains (there were 8 entries out of 500 retrieved from the NCBI database carrying Ile in position 223). The A617T mutation, on the other hand, was not present in any other E. faecalis PBP4 sequence retrieved from the NCBI database. It was also not present in other enterococci like E. termiticus, E. casseliflavus, E. gallinarum, E. caccae, and E. canis. It seems to be a highly conserved position in all PBP4/PBP5 proteins from enterococci. However, this variant is present in two E. faecium PBP5 sequences from early vancomycin-resistant E. faecium in Poland but does not seem to be present in other E. faecium strains.

These data suggest the possibility that β-lactam exposure selects for some lineages of E. faecalis in clinical settings that contain the adenine deletion expressing more PBP4 but may not sufficiently increase the MICs of β-lactams to be easily identified. Combined with a change within the transpeptidase domain, these changes may be sufficient to result in a resistant strain that will be selected by further β-lactam therapy. Because of the proximity of A617T to the active site of the protein, this mutation seems to be quite difficult to evolve. However, the patient from whom this isolate originated received extraordinarily prolonged courses of amoxicillin. The original E. faecalis strain infecting her prosthesis could have already carried the adenine deletion or acquired the deletion during the course of treatment. Persistent amoxicillin selective pressure then may have favored the emergence of the mutant with the A617T substitution, which broke through the amoxicillin treatment.

It remains to be seen whether E. faecalis strains carrying the UP region deletion are becoming more common in the clinical setting, whether this will lead to the emergence of more resistant mutants and to what extent the levels of resistance achieved with these changes will impact the response to treatment of both routine and complicated E. faecalis infections.

## MATERIALS AND METHODS

### Bacterial strains, growth media, chemicals, and microbiological techniques.

The strains used in this study are listed in Table 4. E. faecalis cultures were grown in brain heart infusion (BHI) broth (Fluka) at 37°C. E. coli was grown in Luria-Bertani (LB) broth at 37°C. The concentrations of antibiotics used for selection were as follows: kanamycin, 50 μg/ml (E. coli); spectinomycin, 125 μg/ml (E. coli) or 120 μg/ml (E. faecalis). All antibiotics and chemicals were obtained from Sigma-Aldrich unless otherwise stated. Broth microdilution MIC determinations were carried out by standard methods, except that cells were grown in BHI broth with 2-fold serial dilutions of antibiotics. Plates were incubated at 37°C for 20 h and read by eye. The MIC was defined as the lowest antibiotic concentration inhibiting growth. Testing for the presence of β-lactamase was carried out with nitrocefin disks (Sigma-Aldrich) in accordance with the manufacturer’s instructions.

**TABLE 4:** Bacterial strains and plasmids used in this study

Strain or plasmid	**Description**	**Resistance trait(s)**^a^	**Source**
E. faecalis strains			
LS4828	Penicillin-resistant clinical isolate	Pen^r^ Amp^r^	This study
JH2-2	Penicillin-sensitive isolate	Pen^s^ Amp^s^	19
CH19	β-Lactamase-producing isolate		24
OG1X	Transformation recipient strain	Pen^s^ Spe^s^	25
E. faecium D366		Pen^s^	26
E. coli strains			
DH5α			27
BL21 Star(DE3)			
Plasmids			
pBSU101	Shuttle vector allowing expression of *egfp* in both Gram-negative and Gram-positive organisms	Spe^r^	11
pRIH141	Promoter region of *pbp4* from LS4828 inserted upstream of *egfp* between EcoRI and BamHI sites of pBSU101	Spe^r^	This study
pRIH143	Promoter region of *pbp4* from JH2-2 inserted upstream of *egfp* between EcoRI and BamHI sites of pBSU101	Spe^r^	This study
pET-RP1B	Expression vector allowing amino-terminal His tag fusions	Kan^r^	20
pWP101	*pbp*4*Δ*35 from LS4828 cloned into pET-RP1B for overexpression in E. coli	Kan^r^	This study
pWP102	*pbp*4*Δ*35 from JH2-2 cloned into pET-RP1B for overexpression in E. coli	Kan^r^	This study
pRIH145	pWP102 with Ala617 mutated to Thr	Kan^r^	This study
pRIH146	pWP102 with Val223 mutated to Ile	Kan^r^	This study

a Abbreviations: Pen, penicillin; Spe, spectinomycin; Kan, kanamycin.

Plasmid DNA (pET-RP1B) with the transmembrane deletion-containing versions of E. faecalis PBP4 was transformed into E. coli BL21 Star(DE3) chemically competent cells for expression in accordance with the Champion pET Directional TOPO Expression kit protocol (Thermo Fisher). Starter cultures were grown in LB broth (Sigma) with 50 µg/ml kanamycin overnight at 37°C with shaking at 200 rpm. One liter of LB broth was inoculated with 10 ml of the overnight starter culture and grown to an optical density at 600 nm (OD_600_) of 0.7 to 0.8 at 37°C. The culture was chilled on ice for 1 h and then induced with 0.5 mM isopropyl-β-d-thiogalactopyranoside (IPTG) for 22 h at 28°C. The cells were centrifuged at 4,200 × *g* for 30 min at 4°C in an Allegra X-14R centrifuge (Beckman Coulter, Inc.), resuspended, and washed in 50 mM Tris-HCl (pH 8.0) buffer with 500 mM NaCl (Tris buffer), and cell pellets were stored at −20°C.

### Molecular biology techniques.

DNA-modifying enzymes and kits were purchased from New England Biolabs, unless otherwise noted. Primers were obtained from Sigma. All PCRs were carried out with Phusion High-Fidelity polymerase from New England Biolabs in accordance with the manufacturer’s directions. Plasmid preparations were performed with Qiagen Midi-prep kits. Enterococcal genomic DNA was made with the Qiagen DNeasy kit. Sanger DNA sequencing of PCR products and plasmids was done by GeneWiz (South Plainfield, NJ) and analyzed with DNAStar Lasergene 9 software. The plasmids used in this study are described in Table 4. All of the primers used in this study are described in Table S1 in the supplemental material.

10.1128/mBio.00361-18.1 TABLE S1  Primers used in this study. Download TABLE S1, DOCX file, 0.1 MB. Copyright © 2018 Rice et al. 2018 Rice et al. This content is distributed under the terms of the Creative Commons Attribution 4.0 International license.

### Gene expression studies.

Relevant strains were streaked from frozen stocks onto BHI agar and grown at 37°C. The next day, they were passed again onto BHI agar and incubated overnight. The following morning, 5 ml of BHI broth was inoculated at an OD_600_ of ~0.01 and the bacteria were grown with shaking at 37°C to an *A*_600_ of 0.4 to 0.5. Cells were centrifuged, and cell pellets were frozen at −80°C. RNA was prepared with the Qiagen RNeasy kit after cell lysis with FastPrep Lysing Matrix B glass beads and a Mini-Beadbeater-1 (BioSpec Products). RT-qPCR was carried out on a Bio-Rad CFX96 Real Time System with a Bio-Rad iTaq Universal SYBR green One-Step kit with reactions run with six technical replicates; three biological replicates were prepared. Relative gene expression was calculated by the quantification cycle (*Cq*) method and normalized to the expression of 16S rRNA (17). To compare expression levels, we performed two-tailed unpaired *t* tests with Prism (v7) software (GraphPad Software, Inc.).

### Construction of promoter plasmids and transfer into E. faecalis.

The 200-bp region immediately upstream of *pbp4* from both LS4828 (pRIH141) and JH2-2 (pRIH143) was cloned by PCR into the EcoRI and BamHI sites of pBSU101 (11), producing a transcriptional fusion of *egfp* to the *pbp4* promoter. The sequences of these plasmids were confirmed, and they were transformed into OG1X by electroporation (18). RT-qPCR measuring relative *egfp* levels was carried out as described above to determine if the two different *pbp4* promoters induced different expression levels.

### Cloning of truncated *pbp4* genes for expression in E. coli and construction of point mutations in JH2-2 *pbp4*.

The coding sequences of *pbp4*Δ*35* from both E. faecalis strains JH2-2 (19) and LS4828 were cloned by PCR into the NdeI and XhoI sites of E. coli expression vector pET-RP1B (20). Positive clones were confirmed by Sanger sequencing. Site-directed mutagenesis to alter each of the two amino acids that differ between JH2-2 and LS4828 was done on pWP102 (*pbp4* from JH2-2) by inverse PCR, ligation, and transformation into E. coli DH5α. Clones were confirmed by Sanger sequencing of the coding sequences.

### PBP4 purification for Bocillin FL kinetic studies.

Protein extraction was performed in Tris buffer (pH 8) with 20 mM imidazole, 0.1% Triton X-100, and Halt Protease Inhibitor Cocktail (Thermo Fisher) with 0.1-mm glass beads and bead beating five times for 30 s with 30-s rest intervals. Cell debris and glass beads were removed by centrifugation initially at 4,200 × *g* for 5 min at 4°C and then at 9,000 × *g* for 15 min at 4°C in a Beckman high-speed centrifuge (model SJ2-HS). Clarified, 0.2-µm-filtered (Millipore Express PLUS filter unit) lysate was transferred to a 50-ml conical tube and frozen at −20°C. Purification was performed with Ni Sepharose 6 Fast Flow (GE Healthcare Lifesciences) by batch binding for 1 h at 4°C on a rotator. The unbound protein was removed from the resin by centrifugation at 800 × *g* for 1 min at 4°C. The resin was resuspended by gentle swirling in Tris buffer with 20 mM imidazole, poured into five 10-ml Poly-prep columns (Bio-Rad Laboratories), and allowed to settle. The end cap was then removed, and the flowthrough was collected. Washes were performed with 8 ml of Tris buffer and 20 mM imidazole by inverting the columns to resuspend the resin, allowing it to settle, and then draining it and repeating these steps four times. Elution of the protein (with the His tag intact) was accomplished by a 5-min preincubation of the resin with 8 ml of Tris buffer plus 250 mM imidazole and then gravity flow collection of the eluted protein. The combined elutions were transferred to SnakeSkin 16-mm-diameter dialysis tubing with a 3,500 molecular weight cutoff, (Thermo Fisher) with tobacco etch virus (TEV) protease at a concentration of 1 mg of protease/40 mg of protein and dialyzed for 72 h at 4°C with four changes of 1 liter of 50 mM Tris (pH 8.0)–500 mM NaCl buffer. Cleavage was confirmed by SDS-PAGE, and the final purification of PBP4 from the His-tagged TEV protease was performed with 1.6 ml of cobalt HisPur resin in a Poly-prep column. The purified, untagged protein was collected, buffer exchanged to phosphate-buffered saline (PBS; pH 7.4; Fisher Scientific), and then concentrated to 5 to 10 mg/ml with an EMD Millipore Amicon Ultra-15 (10,000 molecular weight cutoff) centrifugal filter unit. Ten percent glycerol was added, and aliquots of the protein were stored at −20°C. Protein concentrations during purification were estimated by using *A*_280_ measurements with a BioPhotometer (Eppendorf), and final concentrations and purity were determined with the Pierce BCA protein assay kit (Thermo Fisher) and 4 to 12% bis-Tris Nu-PAGE gels (Thermo Fisher) with Precision Plus Protein Standards for purity and size confirmation (Bio-Rad).

### Kinetics of Bocillin FL binding.

Our methods were modified from the work of Hujer et al. (21) and Papp-Wallace et al. (22). All Bocillin FL binding kinetic studies were performed at 37°C in PBS. Bocillin FL titrations with purified PBP4 derived from E. faecalis strains JH2-2, V223I, LS4828, and A617T were performed with 2.78 µg (3.2 µM) of purified protein in 15-µl reaction volumes containing 0 to 320 µM Bocillin FL. These reactions were run for 40 min and stopped with NuPAGE LDS (lithium dodecyl sulfate) sample buffer (Thermo Fisher) and 45 mM DTT (dithiothreitol; Sigma), and the products were incubated at 70°C for 10 min and frozen at −20°C until gel analysis. Sample volumes of 10 µl were loaded into 10% bis-Tris NuPAGE gels and electrophoresed for 70 min at 180 V with chilled 2-(*N*-morpholino)ethanesulfonic acid (MES) running buffer (Thermo Fisher). Prior to imaging, gels were washed twice for 5 min each time with distilled water. The Bio-Rad ChemiDoc XRS+ Imager was used at the trans-UV setting (302-nm filter), and the relative fluorescent intensities were captured by using the autoexposure mode. Analysis was performed with the Lane/Band tools of ImageLab software, and backgrounds were automatically subtracted for each gel. The Michaelis-Menten constants (*K*_*m*_) for each PBP4 enzyme were determined with GraphPad Prism version 5 graphing software with analysis of two independent experiments by using the equation *V*_o_ = *V*_max_([*S*]/[*S*] + *K*_*m*_), where *V*_o_ is the enzyme velocity, *V*_max_ is the maximum enzyme velocity at high substrate concentrations, [*S*] is the Bocillin FL substrate concentration, and *K*_*m*_ is the substrate concentration necessary to achieve half of the maximum enzyme velocity.

The following scheme represents the acylation and deacylation interaction of PBP4 with the fluorescent β-lactam Bocillin FL:

$$\text{PBP}+\beta \text{-lactam}\overset{k_{1}}{\underset{k_{-1}}{⇌}}\text{PBP:}\beta \text{-lactam}\overset{k_{2}}{\to }\text{PBP}-\beta \text{-lactam}\overset{k_{3}}{\to }\text{PBP}+\beta {\text{-lactam}}_{\text{hydrolyzed}}$$

PBP represents the PBP4 enzyme, PBP:β-lactam is the Michaelis complex, PBP–β-lactam is the acylated enzyme, and β-lactam_hydrolyzed_ is the hydrolyzed Bocillin FL product. The reaction rate constants for association and dissociation are *k*_1_ and *k*_−1_, respectively, and *k*_2_ represents the acylation rate constant. The deacylation rate, *k*_3_, is the rate of hydrolyzed product formation, which is very low compared to the acylation rate for this reaction and can be disregarded. The Michaelis constants (*K*_*m*_s) of a β-lactam for PBPs were determined by nonlinear regression analysis of the fluorescence intensity values versus the Bocillin FL substrate concentrations. Acylation rates were determined by reacting 5, 10, 20, 40, 80, and 160 µM Bocillin FL with 2.78 µg of protein and collecting samples at the 0-, 0.5-, 1-, 2-, 4-, and 8-min time points by stopping the reaction on dry ice. We repeated these experiments and analyzed the samples on gels to obtain slopes of the linear time points for each protein to compare binding kinetics. Acylation rates (*k*_2_), representing the formation of a stable PBP-Bocillin FL complex, were determined for the 10 µM Bocillin FL concentration by graphing the mean and standard deviation of SDS-PAGE-based relative fluorescence units over a 4-min time period for two independent experiments.

SimplyBlue SafeStain (Thermo Fisher) was used to stain the gels for confirmation of equal protein loading after the fluorescent imaging of all gels.

### Western blot analysis.

Cell lysates were prepared from 40-ml volumes of BHI broth (Sigma) cultures of cells grown to mid-log phase (OD_600_ of 0.5) and centrifuged to pellet the cells, and then the pellets were stored at −20°C. Cells were lysed by bead beating in 0.6 ml of 10 mM Tris-HCl (pH 7.5)–50 mM NaCl–0.1% NP-40 buffer with Lysing Matrix B tubes and a Mini-Beadbeater-1 (BioSpec Products) three times for 25 s each separated by 5-min intervals on ice. Bacterial membrane samples were prepared from cell pellets from 500-ml BHI cultures grown to an OD_600_ of 0.8. Cells were incubated with 1.4 mg of lysozyme, 244 µg of mutanolysin, and 0.1% DDM (*n*-dodecyl-β-d-maltoside), in 10 mM Tris-HCl (pH 8)–100 mM NaCl for 60 min at 37°C with gentle swirling four to six times during incubation. After 1 h, 28 µl of DNase I (Thermo Fisher) was added and the mixture was incubated at 25°C for 15 min on a platform rotator. The mixture was chilled on ice and then processed by bead beating in Lysing Matrix B tubes as described above. The combined lysates were centrifuged for 10 min at 13,000 × *g* and transferred to 25-ml polycarbonate ultracentrifuge tubes (Beckman Coulter, Inc.), and the membranes were centrifuged in an Optima LE-80K ultracentrifuge with a 60Ti rotor for 1 h at 35,000 rpm and then washed once in PBS (pH 7.4). The membranes were resuspended in 800 µl of PBS, aliquoted, and stored at −80°C. Protein concentrations were determined by Pierce BCA assay, and 20 µg of protein was used per lane for SDS-PAGE and Western blot analysis. All samples were prepared in LDS sample buffer with 50 mM DTT, heated for 10 min at 70°C, loaded into 4 to 12% NuPAGE gels, and then electrophoresed for 40 min at 180 V.

After electrophoresis, proteins were transferred to polyvinylidene difluoride membranes with the iBlot (Invitrogen) dry blotting system, washed with distilled water, and then allowed to dry. Rabbit anti-PBP4 affinity-purified polyclonal antibody (1/3,000 dilution) was procured from New England Peptide with purified E. faecalis PBP4 provided by the W. Peti lab. A bis-Tris detergent-buffered saline solution with Hammarsten casein was used for the blocking and washing steps in accordance with the WesternBreeze blocker/diluent protocol (Thermo Fisher). A goat anti-rabbit IgG horseradish peroxidase-conjugated secondary antibody (Thermo Fisher) was used at a 1/10,000 dilution, and blots were washed and then detected with Clarity Western ECL substrate (Bio-Rad). Digital images and relative intensity units for analysis were obtained with the ChemiDoc XRS+ Imager (Bio-Rad). Duplicate bis-Tris NuPAGE gels were run for molecular weight estimation and protein loading confirmation of Western blot assays with MagicMark XP Western Protein Standard (Invitrogen) and SimplyBlue SafeStain.

### Cloning, expression, and purification of WT and LS4828 PBP4 variants for DSF.

E. faecalis PBP4_36-680_, which lacks the transmembrane domain, was subcloned into the pRP1B expression vector, which includes an N-terminal His_6_ tag and TEV cleavage sequence (20). PBP4 from LS4828 (V223I/A617T) and PBP4 V223I and A617T variants were generated by the QuikChange protocol (Agilent). PBP4 variants were expressed in E. coli BL21(DE3) cells. Cells were grown in Luria broth in the presence of selective antibiotics at 37°C to an OD_600_ of 0.8 to 1.0, and expression was induced by the addition of 0.5 mM IPTG. Proteins were expressed for ~18 h at 18°C prior to harvesting by centrifugation at 6,000 × *g*. Cell pellets were stored at −80°C. For purification, cell pellets were resuspended in lysis buffer (50 mM Tris [pH 8.5], 500 mM NaCl, 5 mM imidazole, 0.1% Triton X-100) and lysed by high-pressure homogenization (Avestin C3 EmulsiFlex). The cell lysate was centrifuged at 47,000 × *g* for 60 min at 4°C, and the clarified supernatant was loaded onto a pre-equilibrated HisTrap HP column (GE). The column was washed with 10 column volumes of wash buffer (50 mM Tris [pH 8.0], 500 mM NaCl, 5 mM imidazole) and eluted with a linear gradient over 20 column volumes into 100% elution buffer (50 mM Tris [pH 8], 500 mM NaCl, 500 mM imidazole).

The fractions containing PBP4 were pooled and dialyzed for 48 h at 4°C with TEV protease to remove the N-terminal His_6_ tag and then subjected to a second Ni-nitrilotriacetic acid purification step to remove the cleaved His_6_ tag, any uncleaved protein, and His_6_-tagged TEV protease. The cleaved PBP4 was then dialyzed for 3 h at 20°C in 1.5 M (NH_4_)_2_SO_4_–10 mM Tris (pH 8.0), after which it was loaded onto a pre-equilibrated HiTrap PhenylHP hydrophobic interaction column (GE Healthcare). Fractions containing PBP4 were pooled, dialyzed against SEC buffer (10 mM Tris [pH 8.5], 300 mM NaCl) for 3 h at 20°C, concentrated, and then purified by size exclusion chromatography pre-equilibrated in SEC buffer (Superdex 200 26/60; GE Healthcare). Fractions containing PBP4 were pooled, concentrated to 1.1 mg/ml, and used immediately for biophysical experiments.

### DSF and modeling.

The WT, LS4828 (V223I/A617T), and A617T versions of PBP4 were purified as described above. Forty microliters of each protein at a concentration of 1.1 mg/ml was added to 10 µl of 5× SYPRO for a final reaction volume of 50 µl. DSF experiments were performed on a CFX96 Real-Time PCR System (Bio-Rad), and the temperature was increased from 4°C and to 80°C in 0.2°C increments. Data were analyzed with the Bio-Rad CFX manager 3.1 software and SigmaPlot. FFAS (23) was used to identify the structures most similar to the sequence of PBP4; 1MWR was selected within FFAS to generate a model of PBP4.
