# Supplementary material for: Structural and Regulatory Changes in PBP4 Trigger Decreased β-Lactam Susceptibility in Enterococcus faecalis
Source: mBio. 2018 Apr 3;9(2):e00361-18. doi: 10.1128/mBio.00361-18 (PMC5885037; doi:10.1128/mBio.00361-18)
Supplement: TABLE S1 [file mbo002183804st1.docx]

Table S1: Primers used in this study

| **Primer** | **Sequence** |
| --- | --- |
| Pbp4-FqPCR | AAGAAGCAAGTCCAGTCAATCT |
| Pbp4-RqPCR | TTGTCTTCTCCCATCCGTAATG |
| 16S-FqPCR | CAAGCGTTGTCCGGATTTATTG |
| 16S-RqPCR | GCACTCAAGTCTCCCAGTTT |
| Pbp4 US-F *Eco*RI | *CCCCCC*gaatTCCTCCTAATAAAAAAC |
| Pbp4 DS-R *BamHI* | ggatccATTCAATAATCCCCTAAC |
| Pbp4 prom FqPCR | GAACCGCATCGAGCTGAA |
| Pbp4 prom RqPCR | TGCTTGTCGGCCATGATATAG |
| Pbp4Δ35 F *Nde*I | CCCCAAGGTcatatgaGTCAATGGCAAGCCAAACAAGAA |
| Pbp4Δ35 R *Xho*I | GTGGTGGTGGTGctcgagTTATTTAATGGTTGCTTC |
| Pbp4 F A617T | ATTG**a**CTGCTAAAACAGGAAC |
| Pbp4 R A617T | GAGAAGTTCGGATTGTAC |
| Pbp4 F V223I | TTTCT**a**TTGATGAAATCAATCAAAAG |
| Pbp4 R V223I | CGCCGAACTTATCACTAAAAGC |

Italic: overhang

Underscore: Restriction site

Bold lower case: Mutagenic base
